# A Lay of Ye Ancient Order of St. John of Jerusalem

**Published:** 1889-06-22

**Authors:** 


### A Lay of ye Ancient Order of St. John of Jerusalem.

Come, all ye dames and demoiselles
Who wish to have a knowledge
Of physical anatomy,
You need not go to college.

Just listen to these lectures on
The human skeleton
Which are given in connection
With the Ambulance of St. John.

You'll hear about the human frame,
That frame of complex nature,
With all its muscles, nerves, and bones,
And their various nomenclature.

Just listen to our lecturer,
And hear how he narrates
About the bones and the "axis"
On which the skull rotates.

You'll learn the number of the bones
In every different part,
And about that organ muscular
Which the vulgar call the heart.

'Bout the "cerebro-spinal system,"
And the ribs—but say "costæ,"
And the "cervix" and the "dorsal,'
And the "lumbar" vertebræ.

And the "scapula" and "clavicle,"
The "os innominata,"
The "cerebellum" and "cerebrum,"
And "medulla oblongata."

The "fibula" and "tibia,"
Eight bones, called wrist, or "carpus,"
The finger bones or "phalanges,"
And the instep bones or "tarsus."

Of "digital pressure" and its work
You will quickly learn the points,
And about "synovial fluid,"
Which lubricates the joints.

To set a fracture you might learn—
I'd advise you not to do it,
The patient might perhaps revive,
And then, no doubt, he'd rue it.

Just diagnose the broken bone
And the signs of dislocating,
The fracture you are sure to know
By "crepitus"—that's grating.

And then the bandaging you'll learn,
And here you must be canny,
For if a "reef" knot you don't tie,
It slips and is a "granny."

Now if you want to learn yet more,
Consult the diagram;
But remember that the climax is—
To pass in the "exam."

